# A Perspective on RNAi-Based Biopesticides

**DOI:** 10.3389/fpls.2020.00051

**Published:** 2020-02-12

**Authors:** Stephen J. Fletcher, Philip T. Reeves, Bao Tram Hoang, Neena Mitter

**Affiliations:** ^1^Centre for Horticultural Science, Queensland Alliance for Agriculture and Food Innovation, University of Queensland, Saint Lucia, QLD, Australia; ^2^Independent Researcher, Canberra, ACT, Australia

**Keywords:** RNAi, biopesticide, topical application, dsRNA, crop protection

## Abstract

Sustainable agriculture relies on practices and technologies that combine effectiveness with a minimal environmental footprint. RNA interference (RNAi), a eukaryotic process in which transcript expression is reduced in a sequence-specific manner, can be co-opted for the control of plant pests and pathogens in a topical application system. Double-stranded RNA (dsRNA), the key trigger molecule of RNAi, has been shown to provide protection without the need for integration of dsRNA-expressing constructs as transgenes. Consequently, development of RNA-based biopesticides is gaining momentum as a narrow-spectrum alternative to chemical-based control measures, with pests and pathogens targeted with accuracy and specificity. Limitations for a commercially viable product to overcome include stable delivery of the topically applied dsRNA and extension of the duration of protection. In addition to the research focus on delivery of dsRNA, development of regulatory frameworks, risk identification, and establishing avoidance and mitigation strategies is key to widespread deployment of topical RNAi technologies. Once in place, these measures will provide the crop protection industry with the certainty necessary to expend resources on the development of innovative dsRNA-based products. Readily evident risks to human health appear minimal, with multiple barriers to uptake and a long history of consumption of dsRNA from plant material. Unintended impacts to the environment are expected to be most apparent in species closely related to the target. Holistic design practices, which incorporate bioinformatics-based dsRNA selection along with experimental testing, represent important techniques for elimination of adverse impacts.

## Background

The demands on global agriculture are expected to escalate in the coming decades, with the population likely to increase to ~9 billion by 2050 (Organisation for Economic Co-operation and Development Staff, and Organisation for Economic Co-operation and Development., OECD environmental outlook to 2050, OECD environmental outlook,OECD, Paris, 2012). Many additional factors are expected to exacerbate the challenges faced by global agriculture, including a move toward greater consumption of more nutritious foods in developing countries with improving economies, decreases in arable land due to urban expansion and land degradation, and perhaps most importantly, adverse effects generated by climate change (FAO, 2009). Climate change associated impacts could include reduced yields due to greater temperatures and extreme weather events, and increased losses owing to expanded and changing ranges of crop pests and pathogens (Chakraborty and Newton, 2011; IPCC, 2014).

Accordingly, sustainable yield increases in the face of the global constraints to production are a necessity. One area where significant productivity gains can be made is limiting crop losses associated with pests and pathogens. Currently, resistant cultivars, chemical pesticides, and integrated management practices are the most efficient methods to respond to biotic challenges. However, since the latter half of the last century, concerns have grown about the use of chemical pesticides, in particular their impacts on human and environmental health, including the lack of differentiation of targets and non-target organisms in their mode-of-action, and widespread development of pesticide resistance. Consequently, the development of innovative and environmentally sustainable approaches to crop protection has become increasingly important.

Among the most notable paradigm shifts in agriculture over the past 50 years is the commercial deployment of genetically modified organisms (GMOs). Crops such as cotton, maize, and soybean have been engineered to be resistant to specific pests and pathogens, yielding staple commodities in many markets. Significant barriers to the uptake of GMOs have however been community acceptance, the cost and time involved, obtaining regulatory approval, and the lack of transformation protocols for many crop species. Herein, we provide a perspective on the development, limitations and risks associated with non-GMO dsRNA-based products which aim to use RNAi to provide protection from crop pests and pathogens in a highly-targeted manner without the need for plant genetic modification.

## The Functional Basis of dsRNA-Based Products: RNAi

RNAi comprises a conserved set of mechanisms that eukaryotes use for regulating RNA transcript abundance. The physiological consequences of RNAi were first identified almost a century ago by Wingard, who observed in tobacco that Tobacco Ringspot Virus infection in the lower leaves was associated with resistance to secondary infection in the upper leaves (Wingard, 1928). The advent of plant and fungi genetic modification lead to observations that the integration of transgenes homologous to endogenous genes sometimes resulted in the suppressed expression of both, a process termed “co-suppression” in plants (Napoli et al., 1990) and “quelling” in fungi (Romano and Macino, 1992). Subsequently, the defensive nature of the process was demonstrated by co-suppressed of viral transcripts, with a transgene expressing a portion of the Tobacco Etch Virus coat protein (CP) able to induce delayed resistance to the virus sequence-specific manner (Lindbo et al., 1993). Fire et al. established that in the nematode *Caenorhabditis elegans*, double-stranded RNA (dsRNA) was a far more potent suppressor of target transcript expression than single-stranded RNA (ssRNA) (Fire et al., 1998). This discovery, for which Fire and co-author Mello were awarded the Nobel Prize, marked the birth of the RNAi revolution.

Practical uses of RNAi were rapidly developed, with transgene-expressed dsRNA employed to induce virus resistance and gene silencing in plants (Waterhouse et al., 1998). Over the following years, other components of the pathway were elucidated. Intermediaries in the form of small ~25 nt antisense RNAs were identified as guides for target RNA degradation (Hamilton and Baulcombe, 1999; Zamore et al., 2000). Dalmay and co-workers showed that the RNA-dependent RNA polymerase RDR6 was recruited to generate dsRNA from target transcripts in plants, leading to a feedback loop of increased small interfering RNA (siRNA) abundance and silencing potential (Dalmay et al., 2000). This process is also evident in fungi and nematodes (Baulcombe, 2004). Other fundamental components then identified included the RNase III domain-containing enzyme responsible for dsRNA cleavage in *Drosophila*, which was termed “Dicer” (Bernstein et al., 2001). Dicer-like genes were also evident in plants and fungi (Jacobsen et al., 1999; Schauer et al., 2002). Members of the conserved Argonaute family were recognized as components of the RNA-induced silencing complex (RISC), which mediated cleavage of the target transcript (Liu et al., 2004; Baumberger and Baulcombe, 2005). Thus, the primary constituents of the RNAi pathway had been identified, with application of the mechanism rapidly advancing across biological fields.

From a risk analysis perspective, the elucidation of many components of the RNAi pathway had important implications for pest and pathogen control applications. RNAi was demonstrated to be highly sequence-specific, allowing for concise dsRNA-directed targeting of transcripts for degradation, however the conserved nature of the pathway among eukaryotes entailed that unintended impacts on non-target organisms (NTOs) could be evident in the presence of the dsRNA if sufficient transcript homology existed.

## RNAi for Protection Against Plant Pests and Pathogens

An important factor that advanced RNAi as a crop protection measure was the observation that the plant's response to virus incursion was functionally related to the response to transgenes [e.g., (Ratcliff et al., 1997; Ruiz et al., 1998)]. The non-cell autonomous nature of RNAi in plants, with local and long distance systemic movement of silencing signals, indicated that co-option of the pathway could provide highly-selective systemic resistance (Tenllado et al., 2004). Though plants had previously been transformed with single-stranded constructs to induce virus resistance, the advantages of expressing dsRNA became clear as the components of the RNAi pathway were characterised. Using a hairpin construct, Wang et al. generated complete resistance to Barley yellow dwarf virus-PAV in transgenic barley, demonstrating the efficiency of the technique in an important commodity crop (Wang et al., 2000).

Though RNAi had previously been used as a tool for examining gene function in insects (Belles, 2010), Baum et al. developed an orally-applied (via artificial diet or transgenic maize) RNAi approach for inducing mortality in the western corn rootworm (*Diabrotica virgifera virgifera* LeConte) *via* targeting various V-ATPase subunits, along with α-tubulin (Baum et al., 2007). In the same year, Mao et al. reported the impairment of growth of cotton bollworm (*Helicoverpa armigera*) by feeding plant leaf material expressing a dsRNA specific to a cytochrome P450 gene (Mao et al., 2007). The approval of the first commercial GMO varieties expressing a dsRNA against an insect pest would not occur until 2017, with Monsanto and Dow's SMARTSTAX PRO maize incorporating a dsRNA against another western corn rootworm gene, *Snf7* (Head et al., 2017). At around the same time, approval was granted for apple and potato expressing dsRNAs for regulation of endogenous gene expression for quality enhancement (Waltz, 2015; Baranski et al., 2019).

In addition to viruses and insects, RNAi has also been adopted for control of many other plant pests and pathogens in a research setting, including bacteria such as *Agrobacterium*, fungi such as powdery mildew, and nematodes such as Root knot nematodes (Rosa et al., 2018). Limitations to the genetic modification approach to crop protection have however been readily apparent for some time, and include low public acceptance in many markets and the inability to genetically transform many crop species (Zotti et al., 2018). Accordingly, much of the recent focus on RNAi for crop protection has shifted toward non-transformative strategies (Dalakouras et al., 2019).

## Development of Topically-Applied RNAi

Functional foliar application of dsRNAs targeting the plant viruses Pepper mild mottle virus (PMMoV), Alfalfa mosaic virus (AMV) and Tobacco etch virus (TEV) was first reported by Tenllado and co-workers in 2001 (Tenllado and Diaz-Ruiz, 2001). In a statement that was to prove prescient, the authors noted that topical application of *in vitro*-expressed dsRNA for protection against plant viruses could be commercially viable provided dsRNA production became inexpensive and an adequate means of delivery was developed (Tenllado and Diaz-Ruiz, 2001). The same authors attempted to reduce the costs of the dsRNA by applying a crude extract of *E. coli* HT115 expressing the same dsRNA fragments used previously, and achieved similarly positive results with viral co-inoculation, though the window of resistance was limited to five to seven days (Tenllado et al., 2003). Additional risks of such an approach relative to the application of purified dsRNA are however evident. These include the potential for toxic fermentation by-products, the presence of selective antibiotics used in growth media, and the uncertain GMO-status of a non-purified product.

Following Tenllado and co-workers' pioneering work, a limited number of reports were evident over the proceeding decade. Gan and co-workers generated dsRNA for topical application against the Sugarcane mosaic virus coat protein using the HT115 system developed earlier by Tenllado (Gan et al., 2010). Lau et al. also used bacterial extracts to generate dsRNA against Cymbidium mosaic virus coat protein (Lau et al., 2014). In more recent years protection from many plant viruses across multiple families has been successfully provided by topical application of dsRNA (Mitter et al., 2017a).

Given the devastation caused by fungal pathogens to crop yield worldwide, the successful topical application of dsRNA to control a fungal infection was significant. Koch et al. showed that *Fusarium graminearum* growth could be inhibited by direct application on detached barley leaves of a dsRNA targeting three CYP450 genes (Koch et al., 2016). Importantly, the ability to inhibit fungal growth spread systemically in the leaf, controlling the pathogen in unsprayed areas. In a recent publication, Höfle et al. demonstrated the length of the sprayed dsRNA impacts on the effectiveness of individual *F. graminearum* CYP gene knockdown, with >1,500bp constructs being much less effective than 200–500 bp constructs (Höfle et al., 2019). By targeting two Dicer-like genes in *Botrytis cinerea*, Wang and co-workers effectively controlled the pathogen on fruit, vegetable and flower surfaces, demonstrating that RNAi could play a role in the post-harvest protection of agricultural produce in addition to pre-harvest protection (Wang et al., 2016). McLoughlin et al. were also able to decrease fungal infection and reduce symptoms in *B. cinerea*, as well as *Sclerotinia sclerotiorum*, *via* foliar application of dsRNA on *Arabidopsis* and *Brassica napus* leaves (McLoughlin et al., 2018).

Relative to viruses and fungi, the development of topical RNAi strategies for protection against arthropod pests has been technically demanding for a range of reasons, including a lack of amplification of the RNAi silencing signal and dsRNA degradation during ingestion (Niu et al., 2018). While oral uptake of dsRNAs targeting critical genes had been shown to induce mortality in some arthropods, transferring delivery from an artificial diet to a topical application strategy has proven difficult. When arthropod pests take up dsRNAs/siRNAs from the plant surface or from internal tissues such as vascular bundles, the abundance of dsRNAs/siRNAs transported to cells where they are effective is comparatively low without protective and uptake enhancement measures being put in place (Niu et al., 2018). Additionally, a study by Biedenkopf et al. indicates that the abundance of RNAi effectors and their ability to induce silencing decreases with distance from the site of exogenous application (Biedenkopf et al., 2019). Interestingly, the authors noted that in the case of barley, the topically-applied dsRNA enters the plant and spreads systemically to leaves, shoots and roots *via* the phloem. It was also evident that the internalised dsRNA was at least partially processed into siRNAs, which could also be detected in distal tissues. These technical barriers have however proven surmountable in some circumstances. The coleopteran Colorado potato beetle was recognised to be highly susceptible to foliar-applied dsRNA, as demonstrated by San Miguel and Scott on potato leaves (San Miguel and Scott, 2016). Non-foliar application methods have also proven successful, with root uptake of target-specific dsRNAs generating mortality in brown planthoppers and Asian corn borers (Li et al., 2015).

Due to circumvention of genetic modification of the host crop, major impediments to adoption of effective topical RNAi approaches are being addressed, including negative public perception of GM-based produce and the inability to transform many important agricultural species. Current research and development of topically-applied RNAi technologies typically focuses on two themes: selecting mortality-maximising target genes specific to a given species, and ensuring topically-applied dsRNAs are sufficiently stable for an optimum protection window. Bioinformatics-based approaches have been used extensively for off-target impact mitigation during the design phase and are discussed below. Stabilization of the dsRNA is a multifaceted issue that is characteristically dependent on the application scenario. Degradation of dsRNA in the environment can occur *via* the actions of ribonucleases and/or UV radiation, both of which are ubiquitous in agricultural settings. The stability of an arthropod-targeting dsRNA should also be sufficient for ingestion, necessitating persistence in non-neutral pH gut conditions prior to delivery to relevant tissues. The use of nanocarriers as components of the delivery system is an option to surmount these hurdles. Nanomaterials have dimensions of less than 100 nm resulting in high surface area to volume ratios, and can be engineered with both protective and slow-release properties for their payloads (Ghormade et al., 2011). Here we present a case study on delivery of dsRNA using clay nanoparticles as carriers, aimed at addressing some of the issues associated with naked dsRNA applications.

## Case Study – Bioclay for Protection and Slow Release of dsRNA on Plant Surfaces

Pioneering work by Tenllado and co-workers on topically-applied RNAi identified the short window of protection offered by a foliar application as an impediment to widespread adoption of the technology (Tenllado and Diaz-Ruiz, 2001). This has also been identified as a key factor in various publications emerging in the last decade on topical application of RNAi [e.g., (Yu et al., 2013; Zotti et al., 2018)].

**Figure 1:**
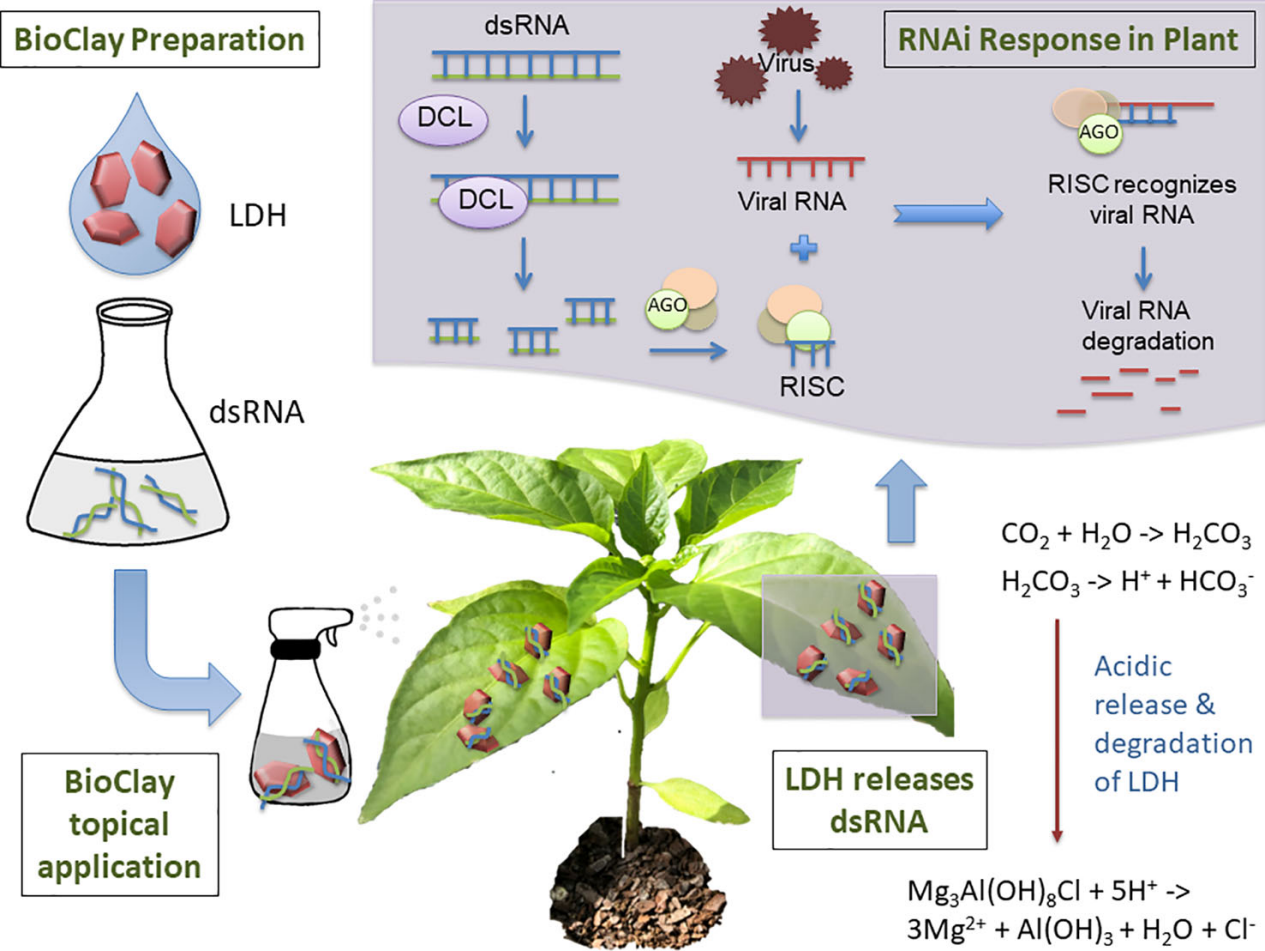
Topical application of BioClay allows for extended RNAi-mediated protection from plant viruses. BioClay is a complex of double-stranded RNA (dsRNA) and layered double hydroxide (LDH). BioClay is prepared by mixing dsRNA and LDH in solution and is applied as a foliar spray. Moisture and carbon dioxide combine to allow acid release of the dsRNA, with LDH degrading to its constituents. The dsRNA can subsequently be taken up by the plant and prime its RNA machinery to degrade homologous viral RNAs. Due to the stabilization and slow release of dsRNA, resistance to the target virus relative to naked dsRNA can be extended from days to weeks.

Mitter et al. explored the use of dsRNA complexed with layered double hydroxide (LDH) nanosheets, termed BioClay, as a spray application (**Figure 1**) (Mitter et al., 2017b). Employing BioClay allowed the window of protection from viral pathogens to be expanded to 20 or more days. Importantly, LDH itself is biocompatible and used in human therapeutics (Del Hoyo, 2007; Kuthati et al., 2015). LDH also safely degrades in the presence of mildly acidic conditions, thus minimising risk of excessive persistence of the dsRNA in the environment. Abating risk while maintaining effectiveness will require similarly novel solutions during the conception of many RNAi-based products, indicating the benefits of risk identification at the earliest stages of development.

## Mitigation and Avoidance of Risks Associated With RNAi-Based Products

**Figure 2:**
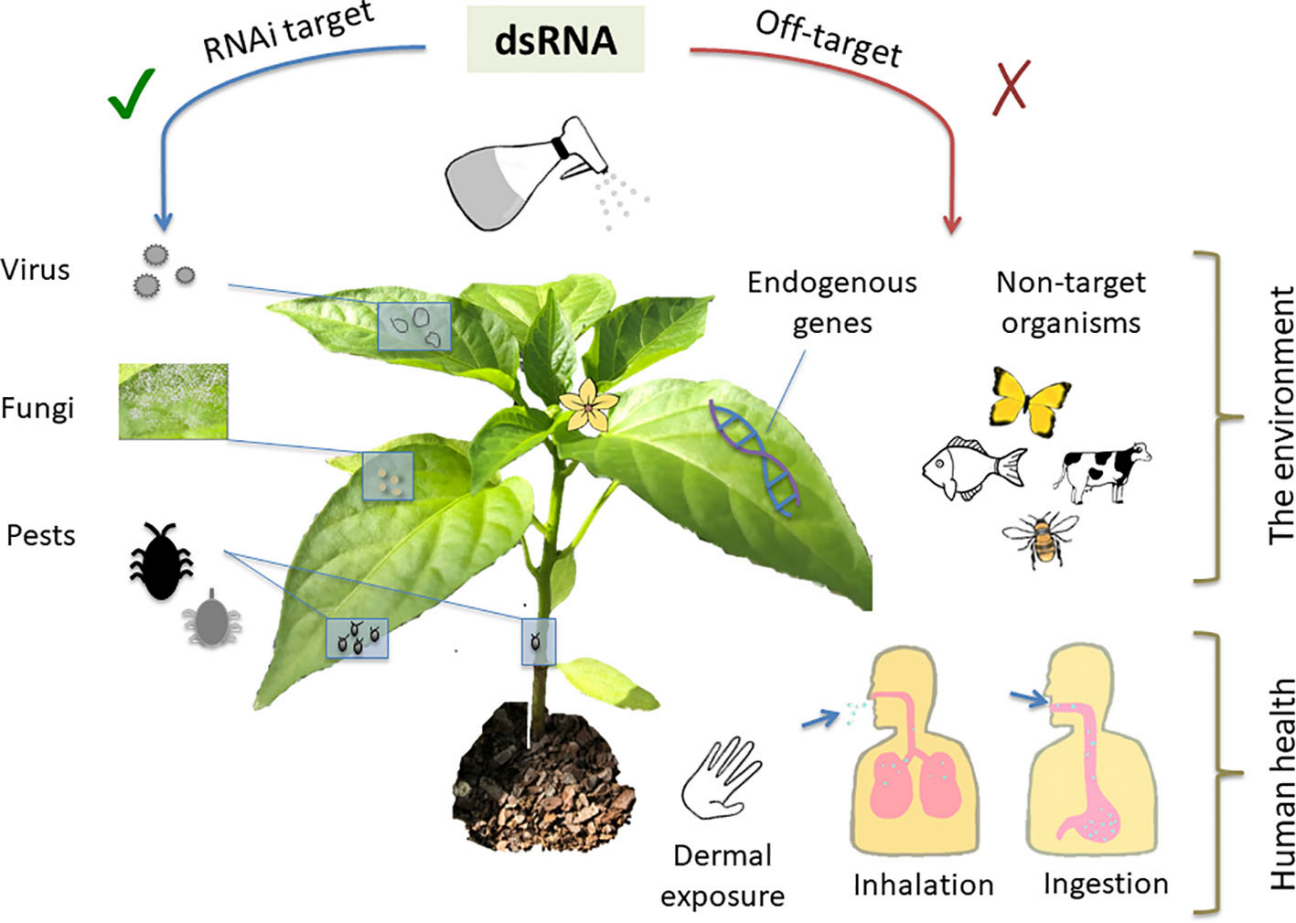
As a crop protection measure, topically-applied dsRNA should be effective against specific pests and pathogens while avoiding unintended adverse consequences. Topically applied double-stranded RNA (dsRNA) can be used to generate resistance to pathogenic viruses and fungi, and pests such as insects. Off-target impacts to be avoided include silencing of crucial host plant and other non-target organism genes. Potential routes of exposure to humans including operators applying the dsRNA along with consumers of treated products could occur *via* dermal exposure, inhalation, and ingestion.

All technologies, whether currently in use or novel, carry a set of risks, which can be either avoided or mitigated through identification, careful planning and design, and safe use practices. Whilst RNAi-based technologies offer clear and obvious safety benefits relative to many existing crop protection products, an analysis of risk is still key to their deployment. The generalized risks associated with environmental application of dsRNA fall into two areas: unintended impacts on human health and unintended impacts to the broader environment (**Figure 2**). When combined with other agents in a formulation, risk analysis of the dsRNA component is less generalizable and should be examined on a case-by-case basis.

## Risks to Human Health

There are three primary routes to human exposure for topically applied dsRNA: ingestion, inhalation, and dermal. For dsRNA to induce a cellular response in humans, it would need to pass through multiple and redundant barriers irrespective of the exposure route. Putative impacts of dsRNA that is internalized could be sequence independent and/or sequence specific. Humans possess an innate immune system that recognises dsRNA in a non-sequence specific manner *via* multiple receptors (DeWitte-Orr et al., 2009; Whitehead et al., 2011). Similar to the situation in other higher eukaryotes, in humans dsRNA can be recognized as a pathogenic signature by the cell, which can then produce an interferon response. Notably, generating such a response *via* inhalation or ingestion is challenging. Humans have a long history of dietary consumption of considerable amounts of dsRNA from virus-infected plant material without any indication of detectable effects, likely due to the rapid degradation of nucleic acids in the stomach in the first instance (Jensen et al., 2013). Few studies have been carried out on the non-sequence specific impacts of dsRNA *via* the inhalation route, as the synthetic dsRNA analogue polyinosinic:polycytidylic acid (poly I:C), which is notable for its ability to induce inflammation and a hypersensitive response, is generally used in mouse and cell line studies as a dsRNA substitute [e.g., (Mahmutovic-Persson et al., 2014)]. The application of poly(I:C) *via* injection under mouse wound scabs has also been shown to induce retinoic acid synthesis and hair follicle regeneration (Kim et al., 2019), though such a route to human exposure would not be considered common occurrence for an RNAi-based biopesticide.

For dsRNA to pose a significant risk to human health in a sequence-specific manner is considered less likely, as a dsRNA would need to be translocated inside a cell rather than to receptors on its surface. The inability to successfully develop RNAi-based therapies indicates the magnitude of the delivery problem (Chen et al., 2018). Instability of non-coding RNAs in biological fluids due to the abundance of endogenous nucleases, and subsequent removal *via* the kidneys, along with inabilities to cross vascular and cellular barriers are cited as key constraints (Chen et al., 2018). Environmental dsRNA without specific protective measures would be similarly affected. An additional constraint for introduced dsRNAs or dsRNA-derived siRNAs is that these sequences must have sufficient homology to endogenous transcripts to induce transcript degradation. Even when this homology is evident, several studies have shown that any impact is likely to be negligible, likely due to the aforementioned delivery constraints. Petrick and co-workers conducted 28-day toxicology trials using dsRNA and siRNAs on mouse models (Petrick et al., 2015). Even with abundant consumption of dsRNAs and siRNAs completely homologous to the mouse vATPase gene, no suppression of gene expression nor any physiological impacts were evident. Indeed, consumption of plant material containing dsRNA capable of generating siRNA homologous to human transcripts is a further indication that sequence-specific impacts are likely unwarranted, at least in the ingestion pathway (Jensen et al., 2013). As noted by Food Standards Australia New Zealand in relation to consumption of dsRNAs from GMOs, “There is no scientific basis for suggesting that small dsRNAs present in some GM foods have different properties or pose a greater risk than those already naturally abundant in conventional foods” (FSANZ, 2013). Formulation of dsRNA-based products with other constituents could however impact human exposure pathways and may require assessment on a case-by-case basis.

## Risks to the Environment

Unintended environmental consequences of RNAi-based products are often case-specific. For example, a beneficial non-target insect that is closely related to a targeted insect pest may be similarly susceptible to environmental RNAi. If there were sufficient dsRNA sequence homology to a key gene, and the beneficial insect had a similar range and feeding patterns, comparable effects would be predicted for mortality. An example of such non-target impacts on related insect species is demonstrated by Baum et al., (2007). Four coleopteran species were fed dsRNA designed to induce mortality. The target Western corn rootworm (WCR) along with the Southern corn rootworm displayed significant mortality upon consumption of a WCR *V-ATPase A*-targeting dsRNA. Colorado potato beetles (CBP) also displayed significant mortality, but the dsRNA was less effective than one directly targeting the CPB *V-ATPase A*. Lastly, the cotton boll weevil suffered no mortality, even with a dsRNA targeting the endogenous CWV *V-ATPase A*. Susceptibility to dsRNA can vary between species, making accurate prediction of gene knockdown a complex issue. In insects, coleopteran species are generally considered the most susceptible to RNAi, with dipterans and hymenopterans sometimes susceptible, and lepidopterans and hemipterans rarely susceptible (Baum and Roberts, 2014; Christiaens et al., 2018).

The development of crop plants expressing dsRNAs against insect pests has led to a number of studies assessing the risks associated with their deployment. Vélez et al. examined the impact on honey bees (*Apis mellifera* L) of maize pollen expressing a dsRNA targeting *V-ATPase A* transcripts of either the target WCR, or the same transcript in the bee itself (Velez et al., 2016). There were no impacts on survival evident at larval or adult stages by either dsRNA, indicating honey bees are not readily susceptible to environmental dsRNA, even with complete sequence homology. Similarly, Tan et al. tested a dsRNA directed against the WCR *DvSnf7* transcript (Tan et al., 2016). Even at higher concentrations than would be present in the field, no impact was evident on honey bee larvae or adults. In a comparable approach to Vélez et al, Pan and co-workers assessed the impact of WCR and endogenous *V-ATPase A* dsRNAs on monarch butterfly (*Danaus plexippus* L) neonates (Pan et al., 2017). No impact on target gene expression or survivability was evident. As with the honey bee, the monarch butterfly did not appear to be susceptible to environmental RNAi. Pan and co-workers also investigated the potential risks of environmental RNAi to the slender springtail (*Sinella curviseta*), again using WCR and endogenous *V-ATPase A* targeting dsRNAs (Pan et al., 2016). Based on artificial diet assays, the authors concluded that adverse impacts to the soil-borne arthropod were negligible. Haller et al. used a WCR *V-ATPase A* dsRNA to determine the responses of two coleopteran ladybird species (*Adalia bipunctata* and *Coccinella septempunctata*) (Haller et al., 2019). As with other coleopteran species, the ladybird species were sensitive to diet-applied dsRNAs, though administered concentrations were much greater than were expected in field conditions. Notably, the degree of negative impacts was associated with the number of homologous matching 21nt sub-sequences for both species, with six matching the *A. bipunctata* transcript and 34 matching the *C. septempunctata* mRNA.

As target sequences become less conserved, the likelihood of inducing deleterious off-target effects is reduced, owing to an inability to produce sufficient off-target homologous siRNAs. Though genetically divergent from the target, one particular off-target organism that is always likely to come into contact with an applied dsRNA is the crop itself. The effectiveness of foliar-applied dsRNA against plant viruses indicates at least a portion of the dsRNA pool is internalized by the plant, which then primes the host RNAi system against viral RNAs. Techniques such as parallel analysis of RNA ends (PARE) have been proposed for identification of endogenous mRNA targets in dsRNA-expressing plants (Casacuberta et al., 2015). Such molecular techniques may also be of use in detecting off-target impacts on crop species following application of RNAi-based biopesticides.

To counter unintended impacts on closely related beneficial species, and indeed any other non-target species the dsRNA may come into contact with, an understanding of the setting in which the RNAi technology will be applied is key, along with careful target sequence selection and subsequent bioinformatics-based design.

## Bioinformatics for Identification and Amelioration of Off-Target Impacts

Degradation of transcripts by the RNAi machinery is directed by siRNAs of ~21–22nt in length, which are generated *via* Dicer from the applied dsRNA. The pool of all possible sense and antisense siRNA sequences derived from a dsRNA can be computationally calculated, allowing for simple identification of homologous non-target transcripts. The degree of homology required to efficiently induce expression knockdown varies, and remains an area of ongoing study. For example, insect feeding studies have shown one or more 19 nucleotide matches between a dsRNA and transcript can reduce transcript expression, which could have deleterious effects if environmental dsRNA were sufficiently abundant (Christiaens et al., 2018).

Taking a precautionary approach to dsRNA design, regions of target genes can be selected to ensure homology to off-target transcripts is minimised, and any contiguous matches above a set limit are identified and avoided (Naito et al., 2005). OfftargetFinder, a web application developed by Good and associates, serves as an example of this approach (Good et al., 2016). Using a database of arthropods and other key species, the software indicates which off-target species have 21nt matches, and allows for the operator to test different regions of a target gene to minimise off-target hits. This application has been used to examine CBP and WCR target genes (*β-actin* and *DvSnf7* respectively) for putative off-target impacts on the lady beetle *Coleomegilla macula* and the red flour beetle *Tribolium castaneum* (Allen, 2017). Another common approach to identify off-target hits has been to use the BLAST search tool. For example, Ulrich et al. employed BLAST to identify contiguous matching regions of 17nt or more between selected off-target insect species and RNAi target genes identified in a large-scale screen of *T. castaneum* (Ulrich et al., 2015).

There are two caveats to the use of the aforementioned approaches, preventing bioinformatics-based selection being the sole arbiter of unintended impacts of a dsRNA. Firstly, an off-target species that possesses a transcript with homology above an arbitrary level may be unaffected for a multitude of reasons; the dsRNA may not be taken up or it may not be transported to a cell where the off-target transcript is expressed (as is likely the case with mammals), transcript degradation may have no impact due to redundancy or other factors, all of which result in no identifiable physiological impact. Consequently, bioinformatics analyses based on homology alone are likely to vastly overestimate the likelihood of off-target impacts, particularly given the abundance of each discrete siRNA generated from a dsRNA is low.

The second deficiency of bioinformatics-based analyses is the lack of genome and transcriptome information currently available for certain beneficial and non-pest species. Fortunately, as genome sequencing costs are rapidly reducing, the public availability of new sequence data that can inform such analyses continues to grow. Focused sequencing may however be required to fill knowledge gaps in specific circumstances.

Notwithstanding these caveats, it is clear a holistic approach to risk avoidance and mitigation has bioinformatics-based design as a component, but is also strongly informed by biological data and an understanding of the biological and ecological systems in which the dsRNA will be deployed.

## Case Study: Regulatory Environment in Australia Pertaining to dsRNA-Based Products for Topical Application to Plants

A critical step in bringing innovative products to market is dialogue between developers and the regulatory authority. In addition to ensuring community and environmental safety, this action provides certainty to developers. Here we provide a case study on the Australian regulators' analysis of where topically-applied RNAi products fit within the existing legal landscape.

Prior to 8^th^ October 2019, topically applied RNAi-based products in Australia were regulated by the Office of the Gene Technology Regulator (OGTR) and the Australian Pesticides and Veterinary Medicines Authority (APVMA). However, this situation changed on 8^th^ October 2019 when approved amendments to the *Gene Technology Regulations 2001* come into effect. The OGTR's *Technical Review of the Gene Technology Regulations 2001* clarifies the regulatory status of organisms developed using a range of new technologies and ensures that new technologies are regulated in a manner commensurate with the risk they pose. In the case of RNA-induced gene silencing pesticides, a new provision clarifies that techniques involving the application of RNA to an organism to temporarily induce RNAi do not constitute gene technology, provided that the RNA cannot be translated into a polypeptide, the organism's genomic sequence cannot be altered as a result, and an infectious agent cannot be produced.

When these conditions are satisfied, the resulting organisms are not GMOs for the purposes of the *Gene Technology Act 2000*. Therefore, RNAi techniques which involve directly applying RNAs to plants for temporarily inducing RNAi have not been subject to regulation by the OGTR since 8^th^ October 2019.

The APVMA will continue to provide regulatory oversight of topically applied RNAi-based products in Australia. Under the *Agricultural and Veterinary Chemicals Code Act 1994*, dsRNA-based products applied topically to protect plants against insect, fungal and viral pests are defined as agricultural chemical products. Data packages in support of the registration of novel agricultural chemical products address, at a minimum, chemistry and manufacture, human health, worker health and safety, environmental fate and toxicity, efficacy and crop safety, and overseas trade. The submitted data for each of these areas should be of sufficient quality for the study to be relied upon for regulatory decision-making. For the APVMA to grant an approval or registration, the APVMA must be satisfied that the safety, trade, and efficacy criteria relevant to the particular active constituent or product are met. Presently, specific guidelines about the types of information that can be submitted to address these criteria for topically applied RNAi-based products are not available; however, the APVMA provides pre-application assistance to prospective applicants, and this service is invaluable for new technologies such as RNA-induced gene silencing pesticides.

## Conclusion

The potential benefits of deploying topically-applied RNAi as a crop protection measure are many, including low toxicity relative to many existing pesticides, species-specificity, and a nominal environmental impact with appropriate dsRNA design. Realizing many of these benefits is however dependent on the development of delivery mechanisms with a similarly light footprint. As with any new technology, there are identified risks that should be avoided in the first instance, and mitigated in the second. Putative unintended consequences primarily relate to impacts on human health and the environment. Given the multiple and redundant barriers to uptake of dsRNA by humans, it appears unlikely that significant deleterious impacts would become evident upon exposure. The ability for dsRNA to rapidly degrade in the environment presumably limits its impact to non-target organisms both at the point of application and post-application. Closely related species to the target species are the most likely to be affected due to their genetic similarity and probable susceptibility to environmental RNAi if they are present in the close vicinity of the application. Bioinformatics-based design of dsRNA sequences to minimise homology with endogenous transcripts in both the host plant and NTOs is an important approach to avoiding and mitigating risks. Limitations of this approach however necessitate it should be part of a suite of tools that help ameliorate any unforeseen consequences for environmental impacts. If conception and development is conducted in a precautionary and rigorous way, RNAi-based products have the ability to revolutionize pest and pathogen management in a safe and effective manner.

## Author Contributions

SF, PR, and NM wrote the manuscript. BH and SF designed the figures. All authors contributed to the conception, revision, editing and approval of the manuscript.

## Funding

This paper was given at the OECD Conference on Regulation of Externally Applied dsRNA-based Products for Management of Pests which took place at the OECD in Paris, France, on 10–12 April 2019, and which was sponsored by the OECD Co-operative Research Programme: Biological Resource Management for Sustainable Agricultural Systems whose financial support made it possible for NM to participate in the workshop.

SF is funded by a Hort Innovation grant, with the Cotton Research and Development Corporation and Nufarm Australia as the co-investors (VG16037). BH is funded by a scholarship from the University of Queensland.

## Disclaimer

The opinions expressed and arguments employed in this paper are the sole responsibility of the authors and do not necessarily reflect those of the OECD or of the governments of its Member countries.

## Conflict of Interest

The authors declare that the research was conducted in the absence of any commercial or financial relationships that could be construed as a potential conflict of interest.
